# The potential association between a new angiogenic marker fractalkine and a placental vascularization in preeclampsia

**DOI:** 10.1007/s00404-021-05966-3

**Published:** 2021-01-26

**Authors:** Grzegorz Szewczyk, Michał Pyzlak, Katarzyna Pankiewicz, Ewa Szczerba, Aleksandra Stangret, Dariusz Szukiewicz, Marta Skoda, Joanna Bierła, Bożena Cukrowska, Anna Fijałkowska

**Affiliations:** 1 Department of General and Experimental Pathology, Medical University of Warsaw, Warsaw, Poland; 2 Department of Pathology, Maria Sklodowska-Curie Institute-Oncology Center, Warsaw, Poland; 3 Department of Obstetrics and Gynecology, Institute of Mother and Child, Warsaw, Poland; 4 Department of Cardiology, Institute of Mother and Child, Warsaw, Poland; 5 Department of Pathology, Children’s Memorial Health Institute, Warsaw, Poland

**Keywords:** Angiogenesis, Preeclampsia, Fractalkine, Placenta

## Abstract

**Purpose:**

Impaired angiogenesis is one of the most common findings in preeclamptic placentas. A new angiogenetic role of fractalkine (CX3CL1) is recently recognized apart from inflammatory activity. In this study, a link between CX3CL1 and the development of placental vasculature in preeclampsia was examined.

**Methods:**

The study comprised 52 women allocated to Group 1 (normotensive, *n* = 23) and Group 2 (preeclampsia, *n* = 29). In each group Doppler parameters, serum levels of CX3CL1, soluble fms-like tyrosine kinase-1 (sFlt-1), and placental growth factor (PlGF) were assessed between 30 and 32 week of pregnancy. After the delivery, placental samples were taken and the vascularization and expression of CX3CR1 receptor were assessed after immunostaining.

**Results:**

CX3CL1 and sFlt-1 serum levels were significantly higher levels in Group 2 vs Group 1, while PlGF serum levels was significantly lower in Group 2. Lower cerebroplacental ratio (CPR) was observed in Group 2. The vascular/extravascular tissue index (V/EVTI) was significantly lower in Group 2, while compared to Group 1, with the lowest value in the fetus growth restriction (FGR) subgroup (0.18 ± 0.02; 0.24 ± 0.03; 0.16 ± 0.02, respectively). The expression of examined CX3CR1 was higher in Group 2, while compared to Group 1, reaching the highest values in FGR subgroup. There was a moderate negative correlation between birth weight, V/EVTI and CX3CL1 serum level and CX3CR1 placental expression in the group of pregnancies complicated with preeclampsia.

**Conclusion:**

The significant underdevelopment of placental vascular network in preeclampsia is associated with the change in the CX3CL1/CX3CR1 system, especially in FGR complicated pregnancies.

## Introduction

The pathogenesis of preeclampsia is still untangled. The most developed theory states that a shallow invasion of extravillous trophoblast towards spiral arteries results in insufficient transformation in high-volume, low-pressure vessels [1]. The concomitant underdevelopment of placental vessels gives severe impairment of both fetal circulation and growth of the fetus [2]. Many factors are recognized to be involved in the process of trophoblast invasion and, among them, the exposure of extravillous trophoblast to hypoxia seems to be the most important [3]. Hypoxia stimulates the expression of transcription factors like hypoxia inducible factor-1α (HIF-1α) and hypoxia inducible factor-2α (HIF-2α). HIF’s are responsible for controlling the expression of genes involved in trophoblast proliferation, differentiation, and invasiveness [4, 5]. Placental angiogenesis is stimulated with a robust increase in a vascular endothelial growth factor (VEGF) release while the source of VEGF is trophoblastic cells. Next, matrix metalloproteases (MMP-2 and MMP-9) and a urokinase plasminogen activator (uPA) are upregulated after HIF’s activation [6]. Both allow for the invasion of trophoblast by the remodelling of an extracellular matrix (ECM).

Interestingly, HIF can be activated in different mechanisms than hypoxia alone. Some cytokines released from immunocompetent cells activate an HIF-1α downstream pathway leading to VEGF transcription and increased angiogenesis in normoxic conditions [7]. E.g., fractalkine (CX3CL1), which can be upregulated by some proinflammatory cytokines such as tumor necrosis factor α (TNFα), interleukin-1 (IL-1), and interferon γ (IFNγ) has the ability to induce HIF-dependent VEGF transcription.

Chemokine CX3CL1 is primarily expressed in a membrane-bound form in neurons and epithelial cells in the lung, kidney, and intestine tissue. Under inflammatory conditions, CX3CL1 is also expressed in endothelial and smooth muscle cells [8]. The CX3CL1 receptor (CX3CR1) is present in natural killer (NK) cells, monocytes, and CD8+ T lymphocytes. Higher levels of soluble CX3CL1 are observed in different inflammatory disorders like asthma, rheumatoid arthritis or osteoarthritis, where CX3CL1 is responsible for chemotaxia of leukocytes, NK cells, and T-cells at the site of inflammation. Therefore, the main role of CX3CL1, apart from chemotaxy, is based on a regulation of the immune system [9]. An interesting specificity of the CX3CR1 is that CX3CL1 is the only ligand and no other receptors for CX3CL1 are recognized.

In a reproductive system, CX3CL1 is present both on the maternal and placental side. The main source of placental CX3CL1 is syncytiotrophoblast, which is constitutively shedded to the maternal circulation and CX3CL1 is released there. In a placenta, CX3CL1 after activation of CX3CR1 is able to induce angiogenesis in the two-step mechanism HIF-1α/VEGF, as well as being able to stimulate integrin-dependent trophoblast migration, the key point in the process of trophoblast invasion towards spiral arteries [10, 11]. As we have shown in our former studies, hypoxia can be an independent stimulator of both CX3CL1 synthesis and CX3CR1 expression in placental perfused lobules [12]. Additionally, other data show that early-onset preeclampsia is characterized by elevated CX3CL1 expression in the placenta [13]. Concerning the angiogenetic potential of CX3CL1, an increase in placental vascularity should be expected. Nevertheless, the other studies showed the defective development of placental vasculature in most cases of preeclampsia. Together with restrained angiogenesis, specific clinical features are present in preeclampsia like decreased blood flow through umbilical arteries and a corresponding brain-sparing effect with increased flow through the fetal brain arteries. Fetal growth restriction (FGR) is complicating about 30% of preeclampsia [14]. Therefore, we examined the potential association of CX3CL1 and known placental angiogenic factors in correlation with the development of placental vasculature and clinical parameters.

## Material and methods

### Patient recruitment

In this prospective, an observational study of a total number of 52 women was included. The study was conducted in accordance with the Helsinki committee requirements protocol and the Ethical Committee of the Institute of Mother and Child approved the study. All patients gave their informed consent before participation in the study. The study was conducted between September 2015 and February 2018. The patients were divided into two groups: Group 1 (*n* = 23)—normotensive women; and Group 2 (*n* = 29)—preeclampsia. Preeclampsia was defined according to the guidelines of the International Society for the Study of Hypertension in Pregnancy (ISSHP) 2014 [15]. Fetal growth restriction (FGR) was defined as birthweight less than 10th percentile.

Inclusion criteria for Group 1 included: over 18 years of age, blood pressure less than 140/90 mmHg during the whole pregnancy, singleton pregnancy, creatinine level less than 1.1 mg/dl. For Group 2: over 18 years of age, diagnosis of preeclampsia based on ISSHP 2014 criteria, singleton pregnancy. Exclusion criteria for both groups were, the presence of fetal anomaly; history of diabetes mellitus; chronic hypertension or connective tissue diseases; HIV positive; HCV positive; toxoplasmosis positive; a use of prophylactic doses of aspirin during pregnancy; smoking during pregnancy. Within Group 2, the FGR subgroup was separated based on criteria as given above. Twelve patients were diagnosed with preeclampsia before 34th week of gestation and seventeen, were diagnosed after the 34th week, however the analysis was performed independently of the time of diagnosis. Clinical characteristics of patients and pregnancy outcome are given in Table 1.

**Table 1:** Maternal characteristics and pregnancy outcomes for the examined cohort

	Group 1 (*n* = 23)	Group 2 (*n* = 29)	*p*	FGR subgroup (*n* = 16)	p
Age (years)	31.0 ± 5.9	33.0 ± 4.1	0.09	33.4 ± 3.9	0.791
Weight (kg)	73.0 (64.6–84.0)	84.0 (74.5–83.0)	0.017	83.5 (69.5–88.5)	0.404
BMI (kg/m^2^)	27.0 (23.8–30.8)	30.5 (27.4–33.6)	0.013	30.4 (26.4–31.6)	0.469
Parity					
Nulliparous	14 [60.8]	18 [62.1]	0.92	10 [62.5]	0.95
Multiparous	9 [39.2]	11 [37.9]		6 [37.5]	
Delivery					
Vaginal	16 [69.5]	6 [20.7]	0.004	2 [12.5]	0.22
Cesarean	7 [30.5]	23 [79.3]		14 [87.5]	
Week of delivery	39 (38–40)	37 (35–37)	< 0.001	35 (32–37)	0.18
Birth weight (g)	3370 ± 592	2335 ± 919	< 0.001	1861 ± 607	< 0.02
Birth weight percentile	44 (25–76)	9 (2–28)	0.002	2 (1–8)	< 0.001
FGR	2 [8.7]	16 [55.2]	< 0.001	16 [100]	N.A

Data presented as mean ± standard deviation if normally distributed data, median (interquartile range) if the distribution was different from normal, and as number [%] if categorical. FGR subgroup was isolated within Group 2 and compared to eutrophic fetuses from Group 2

*BMI* body mass index, *FGR* fetal growth restriction

### Ultrasound examination

Ultrasound examinations with Doppler assessment were performed between the 30th and 32nd weeks of gestation or later if preeclampsia was diagnosed late in the 3rd trimester. The following Doppler parameters were assessed—PI (pulsatility index) of the blood flow in UA (umbilical artery) and MCA (middle cerebral artery), as well as CPR (cerebro-placental ratio) calculated as the rate MCA PI/UA PI. All the parameters were adjusted for the gestational age and given as percentile values according to former studies. [16]

### Blood and placental specimens

Blood samples were taken for assessment of CX3CL1, soluble fms-like tyrosine kinase-1 (sFlt-1), and placental growth factor (PlGF) levels on the same day.

The placenta samples were taken immediately after the delivery of placenta. Each sample was a cubic block of maternal surface placenta, 1 cm each side, taken from the seemingly unchanged region. The tissue fragments were immediately immersed in 10% neutral buffered formalin. After a 24-h period of preservation, the tissue fragments were used to prepare paraffin blocks. A standard histological procedure was used to dehydrate and embed tissue fragments. The blocks were cut on a rotational microtome (Leica Microsystems, UK) into 4 µm-thick tissue sections, subsequently mounted on adhesive glass slides (Menzel GmbH, Germany).

#### Antigen retrieval

Xylene and ethanol passage was used to remove paraffin from the slides. 1% hydrogen peroxide solution was used to block the activity of endogenous peroxidase. Each sample was passed through both xylene and ethanol series for removing paraffins. Next, to prevent endogenous peroxidase activities, samples were incubated with 1% H_2_O_2_. Each sample was washed with a 0.1% tritonx-100 phosphate buffered saline (PBS) for permeabilization and transferred to a citrate buffer solution (pH 6.0) to provide antigen retrieval. Blocking was performed with 2% BSA for 60 min at room temperature.

#### Staining

Selected antigens were labeled with the use of antibodies: anti-CD34 (unconjugated, Dako, Glostrup, Denmark), anti-CX3CR1 (unconjugated, Abcam Inc., Cambridge, MA, USA), anti-cytokeratin 7 (unconjugated, Dako). To visualize presence of unconjugated antibodies Alexa 488 and Alexa 647 were used. DAPI has been used to counterstain nuclei. The staining protocols were based on producer's manuals. We have selected pairs of antigens to be visualized on a single section: CD34 and CX3CR1, and cytokeratin 7 (CK7) and CX3CR1. The slides were investigated and microphotographed on a confocal microscope (FV1000 Olympus).

### The assessment of vascularization index

Identification of the vascular elements in placental sections was performed using an endothelial cell marker—a rabbit polyclonal antibody anti-CD31 (dilution 1:50, ab28364; Abcam). The tissue was incubated with the primary antibody for 30 min. Next, a biotinylated goat anti-rabbit antibody was used as the secondary (Abcam).

Images were acquired with a light microscope equipped with a digital camera and send for analysis to FIJI/ImageJ (Open Source software, National Institute of Health, USA) [17]. The vascular/extravascular tissue index (V/EVTI) was estimated in calibrated areas of the placental sections. Each preparation (paraffin section) underwent three area analyses repeated by two experienced, independent observers. The picture analysis procedure consisted in a measurement of the total vascular area. Consequently, the total lumen area of all types of identified vessels was summed up in both groups. With the aim of minimizing any possible disruption caused by technical errors, especially an unaxial section of the vessel, the lowest value of Ferret’s diameter was accepted as the diameter of a single lumen. Thus, V/EVTI represents the ratio, which reflects intensity of vascularization (vascularization index) and is most closely correlated with the mean density of placental microvessels.

### The assessment of CX3CR1 expression

CX3CR1-immunostained paraffin sections of the placental specimens were subjected to semi-quantitative analysis. The intensity of staining was scored using 0–3 grade and the percentage of positive cells was counted with morphometric software (FIJI/ImageJ) after saving the images obtained from confocal microscopy. The scoring for the intensity was as follows—the score of grade 0 for no reaction or focal weak reaction; grade 1 for intense focal or diffuse weak reaction; grade 2 for moderate diffuse reaction; grade 3 for intense diffuse reaction. Thus, the expression of CX3CR1 was calculated as a multiplication of intensity scoring by the percentage number. The total analyzed area of the single image was 138,246 μm^2^ at 200 × magnification. For each case, five images were recorded and analyzed, two utmost results were rejected and the mean expression value was saved.

### Determination of serum CX3CL1 level

The serum level of CX3CL1 was measured with an enzyme immunoassay kit (Fractalkine/CX3CL1 Human ELISA Kit, Abcam) which uses Sandwich-ELISA as its method. The serum samples were dissolved in an appropriate solution buffer and incubated on microelisa stripplates provided with the kit, pre-coated with an antibody specific to CX3CL1. The optical density (OD) of a colorful enzymatic reaction was measured spectrophotometrically at a wavelength of 450 nm using microplate reader ASYS Biogenet. The minimum detectable dose of CX3CL1 was established as 1.18 pg/ml.

### Determination of serum sFlt-1 and PlGF level

The serum level of sFlt-1 and PlGF was measured with commercially available tests on the fully automated system Cobas^®^ e411 manufactured by Roche. The system is based on electrochemiluminescence immunoassay, with a sandwich immunoassay principle utilizing the streptavidin–biotin method. The following tests were used: Elecsys^®^ sFlt-1 and Elecsys^®^ PlGF (Roche Diagnostics, Poland). The Elecsys^®^ sFlt-1 and PlGF assays limits of detection were 10 pg/ml and 3 pg/ml, respectively. The ratio of sFlt-1/PlGF was calculated for each case.

### Statistical analysis

The Chi-square test was used to analyze categorical data. Quantitative data were presented using mean ± standard deviation (SD) for data with normal distribution and as median; interquartile range (IQR) for data with non-normal distribution. The student’s *T* test was used to compare group locations in case of normal distribution, otherwise the Mann–Whitney *U* test was applied. Kruskal–Wallis test was used to compare subgroups with control group. Spearman correlation analyses were performed to describe the relationship between the biochemical, clinical and morphometrical results. To adjust results for potential confounding factors, a multidimensional GLM analysis was performed. The selection of the best-fit regression models based on the AIC statistic (Akaike information criterion). Regression models were applied to adjust biochemical data with age, weight and BMI (body mass index). The beta coefficients (slopes) were used to measure the effects size and the significance of the factors based on the regression models. In a multidimensional case, the change in the coefficient of determination *r*^2^ before and after fixing the confounding factors was used as the measure of the squared correlation coefficient adjusted for disturbing factors. *p* values less than 0.05 were considered as statistically significant. Data analysis was performed using the SAS statistical package (SAS/STAT rel. 15.1).

## Results

### Serum concentration of angiogenic factors

**Fig. 1:**
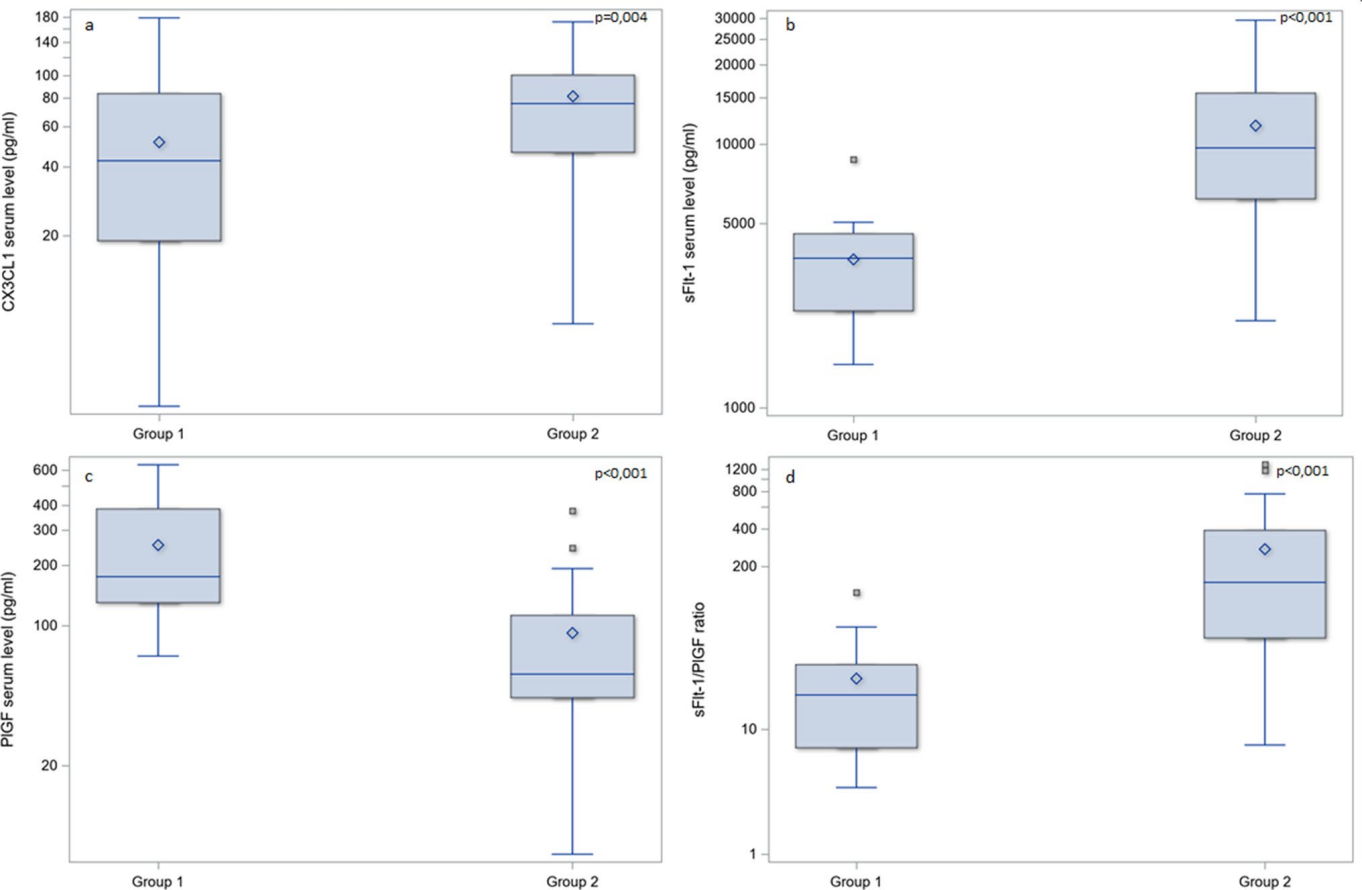
The differences in median serum levels of examined angiogenetic factors between Group 1 (control) and Group 2 (preeclampsia). The box-and-whiskers chart displays basic statistics: mean, median, first and third quartiles as boxes. The extending lines from the boxes indicate variability outside the IQR (interquartile range). The whiskers shows the maximum/minimum observation below/above 1.5 times IQR from the appropriate quartile. Outliers are plotted as single points. CX3CL1 serum level (**a**), sFlt-1 serum level (**b**) and sFlt-1/PlGF ratio (**d**) were significantly higher in preeclamptic group (at *p* = 0.004, *p* < 0.001, *p* < 0.001, respectively), and PlGF serum level (**c**) was significantly lower in preeclamptic group (*p* < 0.001)

Comparing the CX3CL1 levels in the serum of the two examined groups of patients, we revealed significantly higher levels in Group 2 (median 77.8; IQR 46.3–100.9 pg/ml) vs Group 1 (median 41.8; IQR 18.9–83.9 pg/ml), *p* = 0.004. Moreover, the subgroup analysis in preeclamptic women revealed that the highest serum levels were observed in the FGR subgroup (median 95.1; IQR 69.7–127.7 pg/ml) compared to women who delivered newborns with an appropriate weight (median 47.3; IQR 37.9–73.2 pg/ml), *p* = 0.007. The differences in serum levels of sFlt-1 were concordant with previous knowledge. In Group 2 (median 9384.0; IQR 6171.0–15,559.0 pg/ml), the median level of sFlt-1 was significantly higher compared to Group 1 (median 3645.0; IQR 2604.0–4587.0 pg/ml), *p* < 0.001. The median level of PlGF significantly differed between the two examined groups: normotensive women (median 176.0; IQR 130.5–409.4 pg/ml) and preeclamptic women (median 58.1; IQR 44.5–114.9 pg/ml), *p* < 0.001. We noticed a significant difference in the sFlt-1/PlGF ratio between Group 1 (median 18.5; IQR 7.0–31.8) and Group 2 (median 127.0; IQR 51.8–366.4), *p* < 0.001 (Fig. 1). Regression models applied to adjust biochemical data with age, weight and BMI (body mass index) did not reveal any significant correlation.

**Fig. 2:**
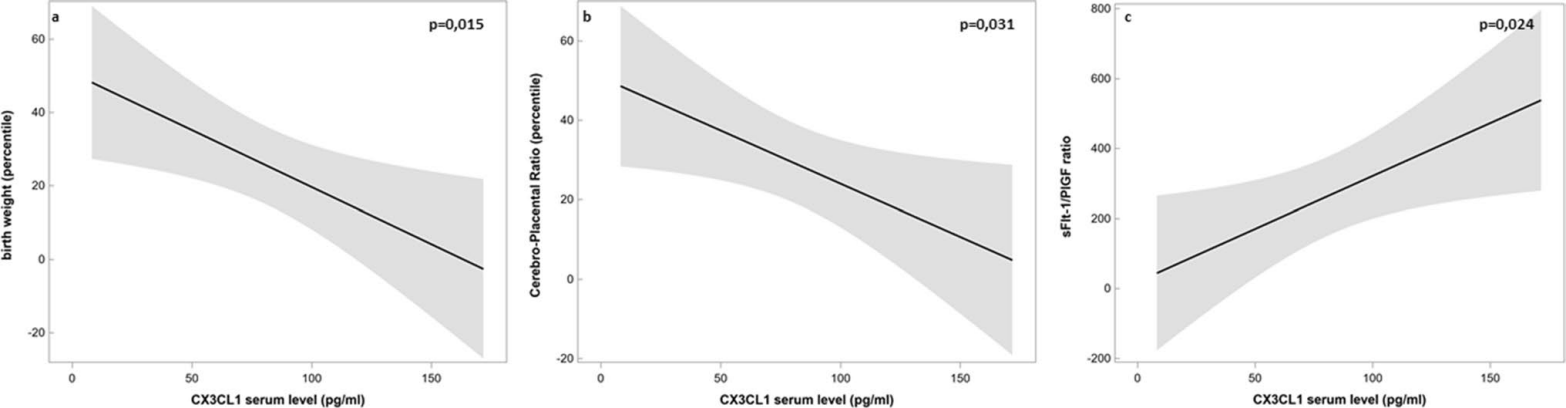
The regression models show significant relations between CX3CL1 serum level and clinical and biochemical parameters in Group 2 (preeclampsia). In Group 1 (control), the analysis did not reveal any significant correlation between CX3CL1 level and examined parameters (not shown on the figure). In the upper right corner of each graph, *p* values are shown. Solid line represents a regression curve, grey zones represent 95% confidence intervals. Based on the regression models, the beta coefficient (slope) between CX3CL1 serum level and birth weight was − 0.31 (*p* = 0.015) (**a**), for cerebro-placental ratio (CPR) the beta coefficient was − 0.27 (*p* = 0.031) (**b**) and for sFlt-1/PlGF ratio the beta coefficient was 3.02 (*p* = 0.024) (**c**). The criterions of the accuracy of the approximation *r*^2^ were 0.25, 0.24 and 0.18, respectively

We also examined the correlation of serum CXCL3 levels with clinical features and other angiogenic factors level. In Group 1 (normotensive women), there was no significant correlation between serum CX3CL1 levels and clinical features in either sFlt-1 or PlGF levels. However, in Group 2, the serum CX3CL1 level was negatively correlated with birth weight (*r* = − 0.54, *p* = 0.003); Doppler parameter—CPR (*r* = − 0.46, *p* = 0.018) and positively correlated with sFlt-1/PlGF ratio (*r* = 0.37, *p* = 0.046). Based on the regression models, the beta coefficient (slope) between CX3CL1 serum level and birth weight was − 0.31 (*p* = 0.015), for cerebro-placental ratio (CPR) the beta coefficient was − 0.27 (*p* = 0.031) and for sFlt-1/PlGF ratio the beta coefficient was 3.02 (*p* = 0.024). The criterions of the accuracy of the approximation *r*^2^ were 0.25, 0.24 and 0.18, respectively (Fig. 2).

### Ultrasound examination results

The median gestational age (weeks of gestation) for ultrasound examination in Group 1 (median 33; IQR 31–34) was not different in comparison to Group 2 (median 31; IQR 31–32), *p* = 0.3 The observed changes in Doppler parameters in Group 2, compared to Group 1, were distinctive for reduced placental blood flow. We observed increased UA PI percentile (median 58.0; IQR 32.0–86.0), decreased MCA PI percentile (mean 27.0 ± 19.9), and lower CPR percentile (median 21.5; IQR 6.0–45.0) in Group 2 (preeclampsia), compared to Group 1 (normotensive), (median 23.0; IQR 17.0–44.0), (mean 45.1 ± 28.7) and (median 61.5; IQR 35.0–79.5), *p* = 0.003, *p* = 0.02, *p* = 0.007, respectively. In Group 2, there were significantly more patients with CPR below 5th percentile (37.9%) in comparison to Group 1 (13%), *p* = 0.04 (Table 2).

**Table 2:** The Doppler indices in examined groups

	Group 1 (*n* = 16)	Group 2 (*n* = 29)	*p*
UA PI	23.0 (17.0–44.0)	58.0 (32.0–86.0)	0.003
MCA PI	45.1 ± 28.7	27.0 ± 19.9	0.02
CPR	61.5 (35.0–79.5)	17.0 (3.0–45.0)	0.007
CPR < 5 percentile	2 [13]	11 [37.9]	0.04

UA PI (pulsatility index in umbilical artery), MCA PI (pulsatility index in a middle cerebral artery), CPR (cerebro-placental ratio). The indices were recalculated to percentile values according to the gestational age. Results are given as mean ± standard deviation if normally distributed data, median (interquartile range) if the distribution was different from normal, and as number [%] if categorical

### The vascularization of placenta

**Fig. 3:**
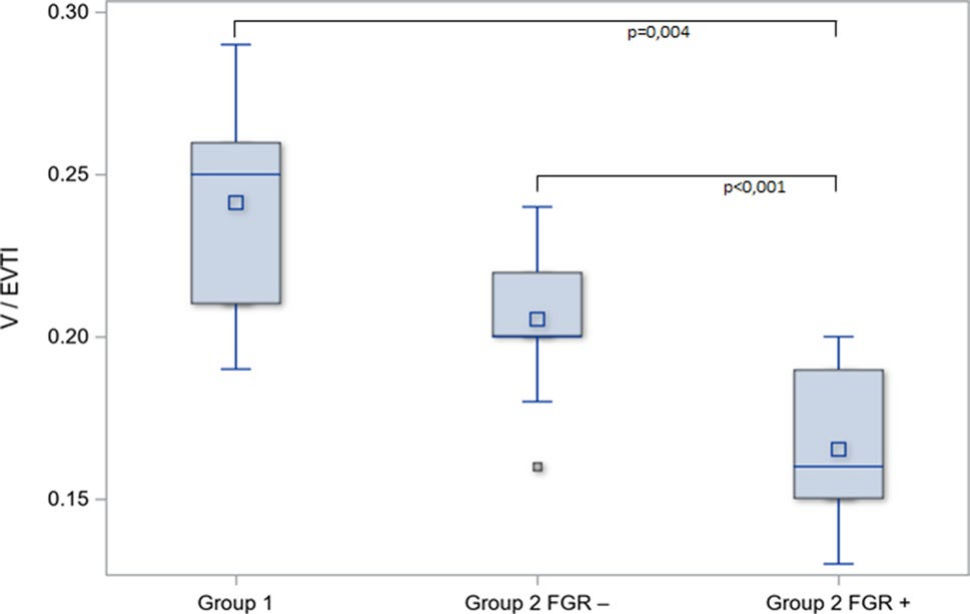
The differences in median levels of vascularization index with FGR subgroup analysis. FGR subgroup within preeclamptic patients (Group 2 FGR+) was compared to eutrophic fetuses from preeclampsia (Group 2 FGR−). Kruskal–Wallis test was employed to check the differences in V/EVTI between Group 1 and subgroups within Group 2 with post hoc analysis. The box-and-whiskers chart displays basic statistics: mean, median, first and third quartiles as boxes. The extending lines from the boxes indicate variability outside the upper and lower quartiles. The whiskers shows the maximum/minimum observation below/above 1.5 times IQR (interquartile range) from the appropriate quartile. Outliers are plotted as single points. The lowest vascularization index (V/EVTI) was observed in placentas examined in pregnancies complicated with preeclampsia and FGR (median 0.16; IQR 0.15–0.19) compared to eutrophic fetuses from preeclampsia (median 0.20; IQR 0.20–0.22), *p* < 0.001

**Fig. 4:**
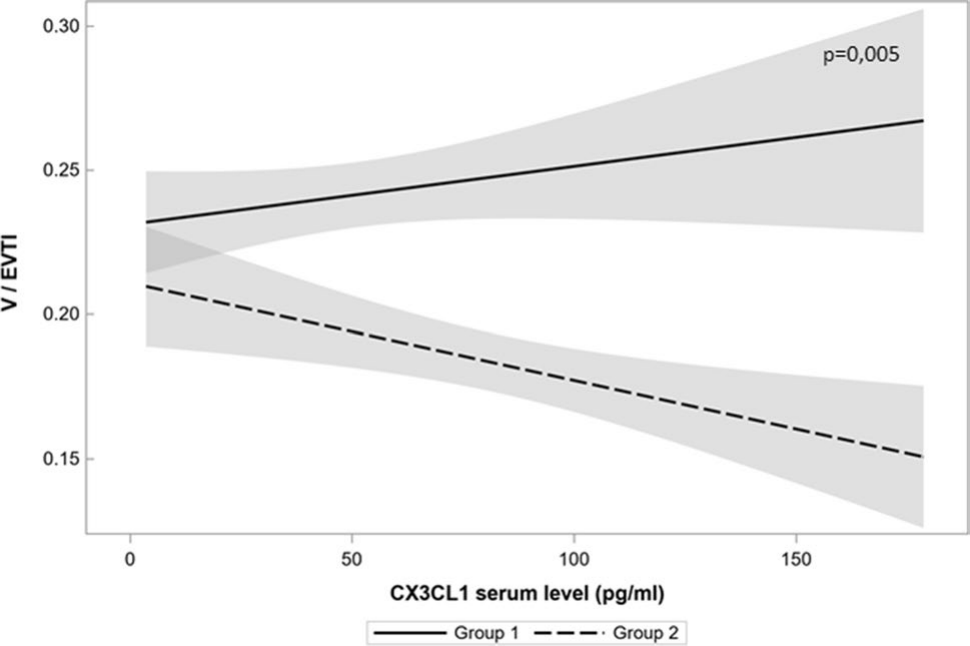
The regression model shows a significant influence of preeclampsia for partial correlations between CX3CL1 serum level and vascularization index (V/EVTI). In the upper right corner of each graph, p value is shown. Solid line represents a regression curve in Group 1 and intermittent line in Group 2, grey zones represent 95% confidence intervals. In the model, the difference between partial correlations due to influence of preeclampsia was statistically significant at *p* = 0.005. Beta coefficient (slope) in the linear relations between CX3CL1 serum level and vascularization index in the absence of preeclampsia was 0.0002 (*p* = 0.169), but in case of preeclampsia, the decrease of the coefficient beta was − 0.00034 (*p* = 0.005). The criterion of the accuracy of the approximation *r*^2^ was 0.59

The vascularization of the placenta was analyzed with the use of V/EVTI as described above. There were significant differences between normotensive and preeclamptic placentas, with reduced vascularization in Group 2 (mean 0.18 ± 0.02) in contrary to Group 1 (mean 0.24 ± 0.03), *p* < 0.001. While analyzing the subgroup of FGR pregnancies within Group 2, V/EVTI was lower in the placentas from FGR fetuses (mean 0.16 ± 0.02) when compared to normal weight fetuses from Group 2 (mean 0.20 ± 0.02), *p* < 0.001 (Fig. 3). In Group 1, a moderate positive correlation between V/EVTI and CPR (*r* = 0.55, *p* = 0.027) and between V/EVTI and birth weight percentile (*r* = 0.46, *p* = 0.026) were observed and in Group 2, a moderate correlation between V/EVTI and CPR (*r* = 0.40, *p* = 0.045), and a strong correlation between V/EVTI and birth weight percentile (*r* = 0.63, *p* < 0.001) were observed. In Group 2, V/EVTI showed also a moderate negative correlation with serum level of CX3CL1 (*r* = − 0.57, *p* = 0.001), which was not observed in Group 1 (*r* = 0.15, *p* = 0.49). Based on the regression model, there has been a confirmed difference between positive correlation in healthy placentas and negative correlation in preeclamptic placentas between CX3CL1 serum level and vascularization index. A multidimensional correlation analysis showed the significant impact of preeclampsia for correlation between V/EVTI and CX3CL1 serum level (*r*^2^ = 0.59, *p* = 0.005). (Fig. 4) There was no significant correlation between V/EVTI and sFlt-1 any of the examined groups.

### The expression of CX3CR1 in placental tissue

**Fig. 5:**
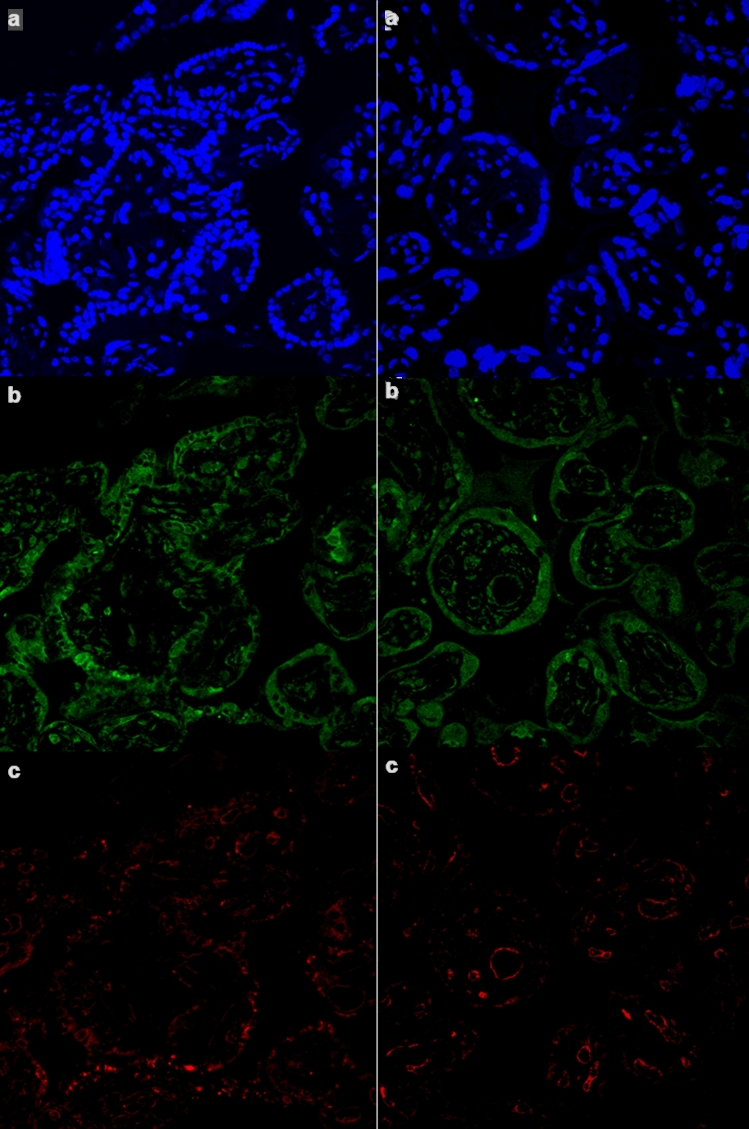
The immunolocalization of CX3CR1 in the placental samples with the confocal microscopy. The left column represents a sample from Group 2 (preeclampsia) and the right column represents a sample from Group 1 (control). Staining with DAPI and localization of cell nuclei (**a**); staining with anti-CK7 antibodies (**b**), localization in trophoblastic cell membrane; staining with anti-CX3CR1 antibodies (**c**), localization in trophoblastic cells in Group 2 and in endothelial cells in Group 1. The significant difference in the expression of CX3CR1 was observed between Group 2 (median 50.0; IQR 30.0–90.0) and Group 1 (median 10.0; IQR 5.0–25.0), *p* < 0.001

**Fig. 6:**
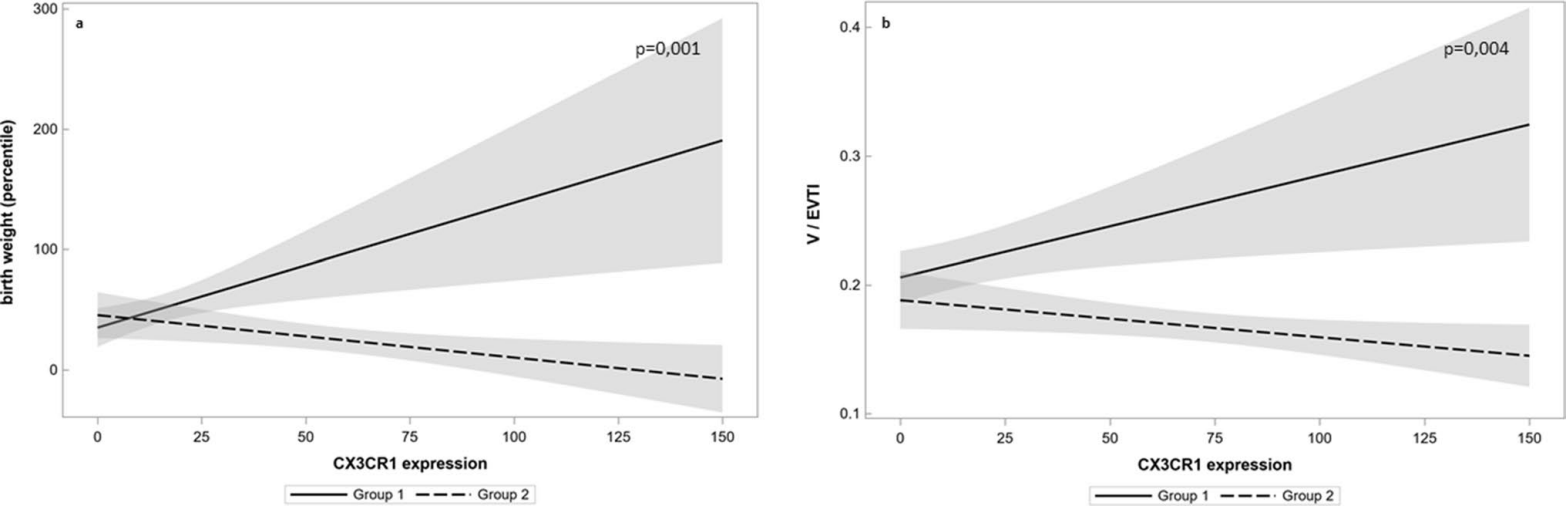
The regression models show a significant influence of preeclampsia for partial correlations between CX3CR1 expression in placenta and birth weight (**a**) and between CX3CR1 expression in placenta and vascularization index (**b**). In the upper right corner of each graph, *p* values are shown. Solid line represents a regression curve in Group 1 and intermittent line in Group 2, grey zones represent 95% confidence intervals. In the model (**a**), the difference between partial correlations due to influence of preeclampsia was statistically significant at *p* = 0.001. Beta coefficient (slope) in the linear relations between CX3CR1 expression and birth weight in the absence of preeclampsia was 1.04 (*p* = 0.007), but in case of preeclampsia, the decrease of the coefficient beta was − 0.35 (*p* = 0.015). The criterion of the accuracy of the approximation *r*^2^ was 0.64. In the model (**b**), FGR was also included. Based on this model, the difference between partial correlations between CX3CR1 expression and V/EVTI due to influence of preeclampsia was statistically significant at *p* = 0.004. Beta coefficient (slope) in the linear relations between CX3CR1 expression and vascularization index in the absence of preeclampsia was 0.00079 (*p* = 0.017), but in case of preeclampsia, the decrease of the coefficient beta was − 0.00029 (*p* = 0.038). The criterion of the accuracy of the approximation *r*^2^ was 0.47

The immunochemical staining for CX3CR1 differed between examined groups. In Group 1, a positive reaction was mostly observed in endothelium, as well as in few circulating macrophages and in Group 2 endothelium reaction was weaker while we observed a positive reaction in syncytiotrophoblast (Fig. 5). Significant differences in the expression of CX3CR1 were observed between Group 1 (median 10.0; IQR 5.0–25.0) and Group 2 (median 50.0; IQR 30.0–90.0), *p* < 0.001. Subgroup analysis revealed a higher expression of CX3CR1 in the preeclamptic placentas from the pregnancies complicated with FGR. The expression of CX3CR1 was significantly higher in the FGR subgroup (median 78.0; IQR 50.0–95.0) compared to eutrophic fetuses within Group 2 (median 40.0; IQR 20.0–40.0), *p* = 0.005. Moreover, the univariate correlation analysis in the control group revealed a moderate positive correlation between CX3CR1 expression and a birth weight percentile (*r* = 0.53, *p* = 0.009) and placental vascularization (*r* = 0.53, *p* = 0.001). The correlation analysis in Group 2 showed the opposite results—there was a negative correlation between CX3CR1 expression and both a birth weight percentile (*r* = − 0.47, *p* = 0.01) as well as vascularization index (*r* = − 0.67, *p* < 0.001). We observed a positive correlation between the expression of CX3CR1 and serum CX3CL1 level in Group 2 (*r* = 0.54, *p* = 0.003). No significant correlation was observed between the CX3CR1 expression and other angiogenic factors levels (sFlt-1 and PlGF) in any of the examined groups. For the correlation between CX3CR1 expression and vascularization index the multivariate regression model was created and showed significant weight of preeclampsia and FGR at *p* = 0.004. Beta coefficient (slope) in the linear relations between CX3CR1 expression and vascularization index in the absence of preeclampsia was 0.00079 (*p* = 0.017), but in case of preeclampsia, the decrease of the coefficient beta was − 0.00029 (*p* = 0.038). The criterion of the accuracy of the approximation *r*^2^ was 0.47. For the correlations between CX3CR1 expression and birth weight, another model was selected, which showed the impact of preeclampsia at *p* = 0.001. Beta coefficient (slope) in the linear relations between CX3CR1 expression and birth weight in the absence of preeclampsia was 1.04 (*p* = 0.007), but in case of preeclampsia, the decrease of the coefficient beta was − 0.35 (*p* = 0.015). The criterion of the accuracy of the approximation *r*^2^ was 0.64 (Fig. 6).

## Discussion

In this study, we examined the potential association between CX3CL1 and the level of development of placental vasculature. However, no consistent associations have been shown under normal conditions (placentas from uncomplicated pregnancies), the meaningful linkage has been noticed in preeclamptic placentas, especially those from pregnancies complicated with fetal growth restriction.

The differences in mean serum levels of examined angiogenetic factors (CX3CL1, sFlt-1, PlGF) between normal and preeclamptic pregnancies were similar to those expected based previous studies. CX3CL1 and sFlt-1 levels were higher in preeclamptic group, and PlGF level was lower significantly. sFlt-1 is recognized as one of the most important biomarkers in prediction of preeclampsia, together with PlGF [18]. sFlt-1 binds VEGF, therefore decreasing its availability for membrane receptors. High serum levels of sFlt-1 in the second trimester are significant for women who develop preeclampsia, with highest levels 5 weeks before clinical onset of preeclampsia [19]. PlGF is very useful in the first trimester in screening for women at risk of preeclampsia, however while measured in third trimester can indicate women who are likely to need a delivery within next 2 weeks, due to preeclampsia [20]. CX3CL1 has not been examined extensively so far and to our knowledge, this is the first study that found a significant difference in serum CX3CL1 levels between healthy and preeclamptic women, as a previous study by Stepanian et al. showed a trend towards elevated levels of CX3CL1 in preeclampsia [21]. This may be explained with the use of different laboratory tests for assessment in these cases. It has been found that the placental secretion of CX3CL1 is significantly increased in cases of severe preeclampsia [13]. However, it should be underlined that another source of CX3CL1 is maternal endothelium and the serum level of CX3CL1 is made of these two elements. The release of CX3CL1 is increased in the third trimester mainly thanks to the activity of metalloproteases and their activity is also increased in cases of preeclampsia [13, 22]. The separate measurement of placental-derived CX3CL1 would give more precise look insight the behaviour of CX3CL1 while preeclampsia development, however it cannot be performed before delivery is finished. Another important issue is the observation of the CX3CL1 variability along with the duration of pregnancy, also if the changes in CX3CL1 can predict the beginning of preeclampsia in any manner.

The only receptor for CX3CL1–CX3CR1 was examined within placental samples and the increased expression was found within syncytiotrophoblast in preeclamptic placenta, especially from pregnancies complicated by FGR. There are a few studies which examine the presence of CX3CR1 in a placenta and their results are not really concordant. The earliest paper from 2008 examined the localization of CX3CR1 in early pregnancy samples and revealed a positive reaction to an endovascular type of extravillous trophoblast and coupled with a weak reaction to the syncytium and cell columns [11]. The suggested role for CX3CR1 was the participation in promotion of trophoblast migration by regulation of adhesion molecules. CX3CL1 by its receptor increased the expression of more than 30 genes encoding proteins involved in process of trophoblast migration including extracellular matrix protein 1, osteopontin, integrin alpha 6, matrix metalloproteinase 12 and integrin beta 5 [23]. Our previous studies performed on placentas from diabetes-complicated pregnancies did not reveal the presence the CX3CR1 on syncytiotrophoblastic cells, only placental endothelial cells were positive for CX3CR1 [24]. The appearance of CX3CR1 on syncytiotrophoblastic cells with concomitant low expression in endothelium in preeclamptic placentas seems to confirm the general pathogenesis of preeclampsia which can be described as a kind of a dysfunctional angiogenesis [25].

The most interesting outcome from the study is the relationship between the CX3CR1 receptors and the development of vascular networks in the subgroup of placentas from preeclampsia and FGR complicated pregnancies, which was confirmed in multidimensional models. CX3CL1 is another proangiogenic factor which acts in two ways: VEGF-dependent and -independent of VEGF pathways. Lee et al. found that CX3CL1-induced angiogenesis was achieved after activation of Raf1/MEK/ERK kinase cascade and PI3K/Akt/eNOS signalling a pathway via CX3CR1 G coupled protein [26]. In vitro experiments showed that CX3CL1 stimulates angiogenesis in a dose-dependent manner and activates HIF-1α pathway and subsequently VEGF-A synthesis in endothelial cells [10]. In animal models, increased CX3CL1 synthesis and local expression of CX3CR1 in endothelium corresponded to increased VEGF-A synthesis and increased local angiogenesis [27]. Increased concentration of CX3CL1 serum levels and an increased expression of CX3CR1 allowed us to expect an increased vascularization of the placenta, while there is a negative correlation between both parameters and V/EVTI. This showed an inactivity of the CX3CL1-CX3CR1 system in the angiogenetic process in this specific subgroup of preeclamptic placentas. This stays in contrary to the control group, where we found a positive correlation between CX3CR1 expression and V/EVTI. It should also be considered that in preeclamptic placentas a linkage between angiogenetic action of CX3CL1 and vessel development is possibly disturbed and not active. A possible autoregulation mechanism between CX3CL1 and CX3CR1 should also be considered as a factor which makes the response to the increased CX3CL1 level by stimulating the expression of its receptor in placental tissue. An autoregulation process concerning macrophages, endothelial cells and others was proposed by many authors [28–30]. An interesting fact, different from previous experiences, is the emergence of CX3CR1 on syncytiotrophoblastic cells in preeclamptic placentas. Looking at this fact, that CX3CL1 is expressed in syncytiotrophoblast and released to the circulation by metalloprotease-dependent shedding, we can support the theory about autocrine mechanism in syncytiotrophoblast, however, this requires further investigation [22]. TNF-α, which is upregulated in preeclampsia, is the strongest stimulator of CX3CL1 synthesis in placenta [13, 31]. The role of CX3CL1 in placenta is dual at least. In the first trimester, CX3CL1 stimulates trophoblast migration through CX3CR1, which is expressed on extravillous trophoblast. In the subsequent stages of the development of a placenta, CX3CL1 is responsible for the interaction between trophoblast and leukocytes on the feto-maternal border. It has been proven that CX3CL1 can stimulate adhesion of THP-1 monocytes and production of inflammatory cytokines like IL8, CCL19, and CCL13 [32, 33]. In preeclampsia, the peripheral subpopulation of “non-classical” CD16+ monocytes, which express CX3CR1, is increased in comparison to uncomplicated pregnancies, and they show up-regulation of some immunomodulating factors like clusterin, lipocalin-2 and leptin receptors [33, 34]. Leptin receptors are, for example, proposed as being responsible for oxidative stress. Therefore, placental expression of CX3CL1/CX3CR1 in preeclampsia can be a part of a developed inflammatory reaction.

The untangled problem is the pathogenesis of a low development of a vascular network in preeclampsia despite an increased angiogenic factor level. Moreover, the clinical findings like birth weight and Doppler parameters were negatively correlated with CX3CR1 expression, confirming disturbed blood flow through placental vessels. Typically, CPR is low in preeclampsia, especially in FGR pregnancies, as placental blood flow through underdeveloped vascular network is performed with a high resistance (and high pulsatility index) and cerebral vessels are dilated for a brain sparing effect, thus giving a low PI. The long-term effect of decreased placental blood flow is presented with a low weight of the fetus. These clinical findings, if set together, give a potential threat to the well-being of the fetus and often push clinicians to a discontinuation of the pregnancy. It seems that in the case of preeclampsia, we have a kind of insensitivity of placental vessels for the angiogenetic action of CX3CL1/CX3CR1, which is observed elsewhere [35, 36]. However, most of the published papers concerned macrophage mediated angiogenesis and were performed as in vitro settings with few exceptions done in animal models [37]. In this case, when we observe an increased expression of CX3CL1/CX3CR1 in placenta and corresponding restrained development of placental vessels, we suggest that the increase in CX3CL1 synthesis is rather the consequence of low angiogenesis in a kind of negative feedback after TNF-α stimulation. The interesting question is the correlation between Doppler parameters and CX3CL1 levels as there is a potential role of CX3CL1 to be another biomarker for clinical decisions, when used together with other biomarkers and clinical data [38].

A strong point of the study is the association of serum, morphometrical and clinical parameters in one setting. Therefore, we were able to show the association between CX3CL1/CX3CR1 pathway and changes in the placental vascular network, correlated with clinical outcome. Summing up, CX3CL1 should be discussed as another player involved in the pathogenesis of preeclampsia, especially in pregnancies complicated by fetal growth restriction.

## References

[1] Fisher SJ (2015). Why is placentation abnormal in preeclampsia?. Am J Obstet Gynecol.

[2] Burton GJ, Jauniaux E (2018). Pathophysiology of placental-derived fetal growth restriction. Am J Obstet Gynecol.

[3] Genbacev O, Zhou Y, Ludlow JW, Fisher SJ (1997). Regulation of human placental development by oxygen tension. Science.

[4] Cowden Dahl KD, Fryer BH, Mack FA, Compernolle V, Maltepe E, Adelman DM, Carmeliet P, Simon MC (2005). Hypoxia-inducible factors 1alpha and 2alpha regulate trophoblast differentiation. Mol Cell Biol.

[5] Baines KJ, Renaud SJ (2017). Transcription factors that regulate trophoblast development and function. Prog Mol Biol Transl Sci.

[6] Pringle KG, Kind KL, Sferruzzi-Perri AN, Thompson JG, Roberts CT (2010). Beyond oxygen: complex regulation and activity of hypoxia inducible factors in pregnancy. Hum Reprod Update.

[7] Patel J, Landers K, Mortimer RH, Richard K (2010). Regulation of hypoxia inducible factors (HIF) in hypoxia and normoxia during placental development. Placenta.

[8] White GE, Greaves DR (2012). Fractalkine: a survivor’s guide: chemokines as antiapoptotic mediators. Arterioscler Thromb Vasc Biol.

[9] D’Haese JG, Friess H, Ceyhan GO (2012). Therapeutic potential of the chemokine-receptor duo fractalkine/CX3CR1: an update. Expert Opin Ther Targets.

[10] Ryu J, Lee C-W, Hong K-H (2008). Activation of fractalkine/CX3CR1 by vascular endothelial cells induces angiogenesis through VEGF-A/KDR and reverses hindlimb ischaemia. Cardiovasc Res.

[11] Hannan NJ, Jones RL, White CA, Salamonsen LA (2006). The chemokines, CX3CL1, CCL14, and CCL4, promote human trophoblast migration at the feto-maternal interface. Biol Reprod.

[12] Szukiewicz D, Kochanowski J, Mittal TK, Pyzlak M, Szewczyk G, Cendrowski K (2014). CX3CL1 (fractalkine) and TNFα production by perfused human placental lobules under normoxic and hypoxic conditions in vitro: the importance of CX3CR1 signaling. Inflamm Res Off J Eur Histamine Res Soc Al.

[13] Siwetz M, Dieber-Rotheneder M, Cervar-Zivkovic M, Kummer D, Kremshofer J, Weiss G, Herse F, Huppertz B, Gauster M (2015). Placental fractalkine is up-regulated in severe early-onset preeclampsia. Am J Pathol.

[14] McKinney D, Boyd H, Langager A, Oswald M, Pfister A, Warshak CR (2016). The impact of fetal growth restriction on latency in the setting of expectant management of preeclampsia. Am J Obstet Gynecol.

[15] Tranquilli AL, Dekker G, Magee L, Roberts J, Sibai BM, Steyn W, Zeeman GG, Brown MA (2014). The classification, diagnosis and management of the hypertensive disorders of pregnancy: a revised statement from the ISSHP. Pregnancy Hypertens.

[16] Baschat AA, Gembruch U (2003). The cerebroplacental Doppler ratio revisited. Ultrasound Obstet Gynecol Off J Int Soc Ultrasound Obstet Gynecol.

[17] Schindelin J, Arganda-Carreras I, Frise E (2012). Fiji: an open-source platform for biological-image analysis. Nat Methods.

[18] Lecarpentier E, Tsatsaris V (2016). Angiogenic balance (sFlt-1/PlGF) and preeclampsia. Ann Endocrinol.

[19] Levine RJ, Maynard SE, Qian C (2004). Circulating angiogenic factors and the risk of preeclampsia. N Engl J Med.

[20] Chappell LC, Duckworth S, Seed PT (2013). Diagnostic accuracy of placental growth factor in women with suspected preeclampsia: a prospective multicenter study. Circulation.

[21] Stepanian A, Benchenni S, Beillat-Lucas T (2009). Search for an association between V249I and T280M CX3CR1 genetic polymorphisms, endothelial injury and preeclampsia: the ECLAXIR study. PLoS ONE.

[22] Siwetz M, Blaschitz A, Kremshofer J, Bilic J, Desoye G, Huppertz B, Gauster M (2014). Metalloprotease dependent release of placenta derived fractalkine. Mediat Inflamm.

[23] Hannan NJ, Salamonsen LA (2008). CX3CL1 and CCL14 regulate extracellular matrix and adhesion molecules in the trophoblast: potential roles in human embryo implantation. Biol Reprod.

[24] Szukiewicz D, Kochanowski J, Pyzlak M, Szewczyk G, Stangret A, Mittal TK (2013). Fractalkine (CX3CL1) and its receptor CX3CR1 may contribute to increased angiogenesis in diabetic placenta. Mediat Inflamm.

[25] Redman CWG, Staff AC (2015). Preeclampsia, biomarkers, syncytiotrophoblast stress, and placental capacity. Am J Obstet Gynecol.

[26] Lee S-J, Namkoong S, Kim Y-M, Kim C-K, Lee H, Ha K-S, Chung H-T, Kwon Y-G, Kim Y-M (2006). Fractalkine stimulates angiogenesis by activating the Raf-1/MEK/ERK- and PI3K/Akt/eNOS-dependent signal pathways. Am J Physiol Heart Circ Physiol.

[27] Zhang J, Yang W, Luo B, Hu B, Maheshwari A, Fallon MB (2012). The role of CX_3_CL1/CX_3_CR1 in pulmonary angiogenesis and intravascular monocyte accumulation in rat experimental hepatopulmonary syndrome. J Hepatol.

[28] Mizutani N, Sakurai T, Shibata T, Uchida K, Fujita J, Kawashima R, Kawamura YI, Toyama-Sorimachi N, Imai T, Dohi T (2007). Dose-dependent differential regulation of cytokine secretion from macrophages by fractalkine. J Immunol Baltim Md 1950.

[29] Morimura S, Sugaya M, Sato S (2013). Interaction between CX3CL1 and CX3CR1 regulates vasculitis induced by immune complex deposition. Am J Pathol.

[30] Chandrasekar B, Mummidi S, Perla RP, Bysani S, Dulin NO, Liu F, Melby PC (2003). Fractalkine (CX3CL1) stimulated by nuclear factor kappaB (NF-kappaB)-dependent inflammatory signals induces aortic smooth muscle cell proliferation through an autocrine pathway. Biochem J.

[31] Rambaldi MP, Weiner E, Mecacci F, Bar J, Petraglia F (2019). Immunomodulation and preeclampsia. Best Pract Res Clin Obstet Gynaecol.

[32] Siwetz M, Sundl M, Kolb D, Hiden U, Herse F, Huppertz B, Gauster M (2015). Placental fractalkine mediates adhesion of THP-1 monocytes to villous trophoblast. Histochem Cell Biol.

[33] Nonn O, Güttler J, Forstner D, Maninger S, Zadora J, Balogh A, Frolova A, Glasner A, Herse F, Gauster M (2019). Placental CX3CL1 is deregulated by angiotensin II and contributes to a pro-inflammatory trophoblast-monocyte interaction. Int J Mol Sci.

[34] Alahakoon TI, Medbury H, Williams H, Fewings N, Wang XM, Lee VW (2019). Characterization of fetal monocytes in preeclampsia and fetal growth restriction. J Perinat Med.

[35] Kumar AHS, Martin K, Turner EC, Buneker CK, Dorgham K, Deterre P, Caplice NM (2013). Role of CX3CR1 receptor in monocyte/macrophage driven neovascularization. PLoS ONE.

[36] Sun X-T, Zhang M-Y, Shu C, Li Q, Yan X-G, Cheng N, Qiu Y-D, Ding Y-T (2005). Differential gene expression during capillary morphogenesis in a microcarrier-based three-dimensional in vitro model of angiogenesis with focus on chemokines and chemokine receptors. World J Gastroenterol.

[37] Park Y, Lee J, Kwak J-Y, Noh K, Yim E, Kim H-K, Kim YJ, Broxmeyer HE, Kim J-A (2018). Fractalkine induces angiogenic potential in CX3CR1-expressing monocytes. J Leukoc Biol.

[38] Hendrix M, Bons J, van Haren A, van Kuijk S, van Doorn W, Kimenai DM, Bekers O, Spaanderman M, Al-Nasiry S (2019). Role of sFlt-1 and PlGF in the screening of small-for-gestational age neonates during pregnancy: a systematic review. Ann Clin Biochem.

